# An isolated congenital absence of nasal columella: A case report and review of literature

**DOI:** 10.1016/j.ijscr.2019.04.034

**Published:** 2019-04-22

**Authors:** Bareqa Salah, Isam Bsisu, Osama Sarhan, Zayed Al-Zu’bi, Ahmad Suleihat

**Affiliations:** a Jordan University Hospital, Department of General Surgery, Jordan; b Jordan University Hospital, Jordan; c Jordan University Hospital, Department of Oral and Maxillofacial Surgery, Jordan

**Keywords:** Congenital, Absence, columella

## Abstract

**Introduction:**

The nasal columella is an important aesthetic and functional entity of the midface. Congenital absence of the nasal columella as an isolated anomaly is extremely rare.

**Presentation of case:**

We present a case of total congenital aplasia of the nasal columella in a 3-month-old female infant. The deformity was not associated with any other anomalies and with no obvious underlying cause.

**Discussion:**

Isolated congenital absence of the nasal columella is an extremely rare anomaly and its etiology is still unknown. Surgical reconstruction of absent nasal columella is challenging as a result of its complex anatomy, and different surgical techniques have been described in the literature to reconstruct the nasal columella with varying advantages and disadvantages.

**Conclusion:**

The variety in available surgical techniques for the reconstruction of nasal columella necessitates a thorough preoperative evaluation in order to choose the most suitable surgical technique and achieve the best outcome, both functionally and esthetically.

## Highlights

• The absence of nasal columella has functional and esthetic consequences.
• The etiology of isolated congenital absence of nasal columella is still unknown.
• Reconstruction of absent columella is challenging due to its complex anatomy.

## 1 Introduction

Nasal columella is the tissue which connects the nasal tip to the nasal base and separates the nares [1]. The columella has a major esthetic and structural role at the inferior margin of the nasal septum, and its absence has both functional and esthetic consequences [2]. The absence of the columella generally occurs due to infection, trauma, malignancy, surgical excision of tumors, or may be congenital [3]. We present a case of a 3-month-old female infant who presented with isolated congenital absence of nasal columella. This work has been reported in line with the SCARE criteria [4].

## 2 Case presentation

**Fig. 1:**
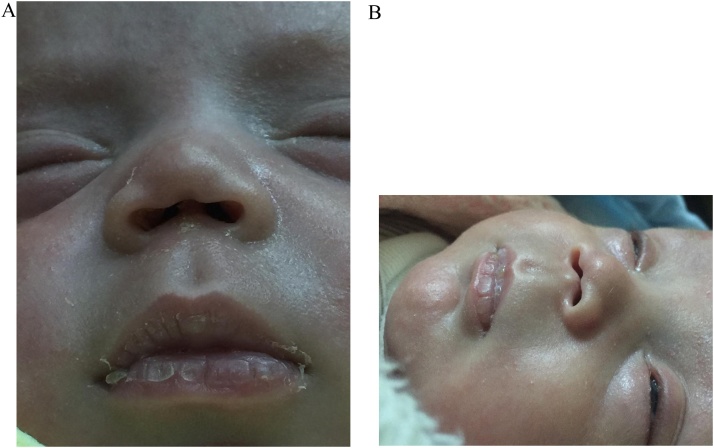
Inferior (A) and lateral (B) views showing total absence of the nasal columella from the nasal tip down to the root of the philtrum, involving the medial crura of the ala cartilage.

An 89-day old female infant, presented to our clinic with absent nasal columella since birth (Fig. 1). The patient was a product of a normal vaginal delivery (NVD) of a preterm (27 weeks) pregnancy, with birth weight of 1.1 kg. The patient was admitted to the NICU for prematurity and respiratory distress, and was discharged after 70 days. Currently, she has no difficulty in breathing nor feeding. The patient has family history of congenital heart disease of her uncle who is currently 10 years old and is doing well and both of her elder brother and sister are medically free. Moreover, the mother denied radiation exposure or utilization of any medications during pregnancy or breast feeding. The patient has no previous history of trauma, malignancies or infections, and there was no consanguinity between the parents.

The physical examination revealed that her growth chart (weight, height, and head circumference) is at the 30^th^ percentile according to the CDC growth chart for females below 36 months. A total absence of the nasal columella from the nasal tip down to the root of the philtrum, involving the medial crura of the ala cartilage. Surrounding structures such as the septum, nose, and upper lip are normal. The rest of her physical examination was entirely normal.

The laboratory investigations, chest x-ray, echocardiogram, and ultrasound of the abdomen were all unremarkable. After discussing the treatment options with the patient’s parents, they preferred the option of having the newly introduced 2-stage reconstruction of the columella described by Pan et al. [5] after the age of one year. The first stage will involve bilateral nasal sill flaps that will be elevated and mobilized medially to create the new columella, and in the second stage we will insert an auricular composite graft to provide support where a portion of the caudal septum was missing, and to complete the new columellar reconstruction [5].

## 3 Discussion

Congenital absence of the nasal columella is an extremely rare anomaly [2,3,[5], [6], [7], [8], [9], [10]]. To the best of our knowledge, this is the youngest patient in reported literature to present with isolated congenital absence of the nasal columella. The reason for the rarity of this defect may be that the anomaly is an inconspicuous one; as a result, the patients often do not seek medical attention until late childhood or adolescence.

Our review of the literature indicated that Jacobs et al. [10] first reported a case of a young male patient in 1984 with isolated absence of the columella. Another report of isolated anomaly from Lewin et al. [9] who in 1988 reported three cases; one in a male patient, and the other two were twin female patients.

The etiology of isolated congenital absence of nasal columella is still unknown [3]. The embryonic development of the nose takes place between the 3^rd^ and 10^th^ weeks of gestation [6]. The medial nasal processes fuse in the midline with the frontal prominence, resulting in the formation of the frontonasal process which gives origin to the columella [6,11]. This supports the assumption that teratogen introduced during the period of columellar development might selectively arrest the cellular penetration and impede chondrification of the nasal columella [3,6].

Surgical reconstruction of absent nasal columella is challenging as a result of its complex anatomy. The columella is composed of 3 proportionate and harmonious segments: the anterior lobular segment, the narrow intermediate segment, and the flared basal segment [12]. The medial footplates of the columella and the caudal septum contribute to the projection of the nasal tip [5], which necessitates delicate correction in order to achieve the best outcome, both functionally and esthetically.

A thorough preoperative evaluation is necessary in order to assess the subunits and the layers involved, and discuss different reconstructive options [12]. Esthetic consideration of donor site, such as skin texture color, and contour, alongside with donor site selection with its potential morbidity are essential, in order to obtain the optimum results [2]. When finalizing the preoperative assessment, the patient’s parents must be counseled regarding the expected esthetic results and the expected recovery time, in addition to the addressing their concerns regarding patient care during and after that perioperative and recovery period [12].

Different surgical techniques have been described in the literature to reconstruct the nasal columella. Chondrocutaneous auricular composite grafts are convenient for providing cartilaginous support non-hair-bearing skin that is well-matched in color and texture [5]. However, the utilization of this method is limited by the fact that graft survival is influenced by vascular bed of the recipient site [5,12,13]. On the other hand, the utilization of local random pattern flaps and regional grafts lessens the concern for a well-vascularized bed of the recipient area. However, both techniques may have possible disadvantages, such as requiring multiple operative stages, donor site distortion, and transferring of hair-bearing skin [12]. The introduction of free tissue transfer has extended the spectrum of reconstructive procedures for columella reconstruction. Composite flap procedures, such as preauricular flap, prefabricated retroauricular flap, and first webspace of the foot flap, are promising alternatives that succeeded in providing both external coverage and internal support. However, the advanced technical aspects and longer procedure times are the main disadvantages [12]. The newly introduced 2-stage reconstruction for isolated columellar defects [5] consists of modified Cronin procedure in the first stage that is similar to the one described by Demir et al. [14,15] in order to form the basic external columellar outline using bilateral nasal sill flaps that are advanced anteromedially, which will provide a well-vascularized bed for the second stage. In the second stage, we may place an auricular composite graft from the helical root in order to provide structural support, and to augment the anticipated convex contour of the new columella while conserving adequate tip projection [5,12].

In conclusion, the absence of nasal columella has significant esthetic and structural consequences. Over the years, a large variety of surgical techniques have been described for the reconstruction of the columella, for which preoperative evaluation assessing the subunits and the layers involved is essential in order to discuss different reconstructive options and choose the most suitable surgical technique for the patient, after ruling out other congenital anomalies.

## Conflicts of interest

The authors have no conflict of interest to declare.

## Sources of funding

No funding.

## Ethical approval

This article was approved by the ethics committee and IRB of the University of Jordan and Jordan University Hospital.

## Consent

Written informed consent was obtained from the patient’s parents for publication of this case report and accompanying images. A copy of the written consent is available for review by the Editor-in-Chief of this journal upon request.

## Author’s contribution

Bareqa Salah: study consent, design, writing of the paper and revision of final manuscript.

Isam Bsisu: study design, writing of the paper and revision of final manuscript.

Osama Sarhan: study consent, design, and revision of the final manuscript.

Zayed Al-Zu’bi: study consent, design, and revision of the final manuscript.

Ahmad Suleihat: study consent, design, and revision of the final manuscript.

## Registration of research studies

Not applicable to our manuscript.

## Guarantor

Bareqa Salah.

## Provenance and peer review

Not commissioned, externally peer-reviewed.
